# Measuring Water Quantity Used for Personal and Domestic Hygiene and Determinants of Water Use in a Low-Income Urban Community

**DOI:** 10.3390/ijerph192315656

**Published:** 2022-11-25

**Authors:** Rebeca Sultana, Nazmun Nahar, Stephen P. Luby, Sayeda Tasnuva Swarna, Emily S. Gurley, Charlotte Crim Tamason, Shifat Khan, Nadia Ali Rimi, Humayun Kabir, Md. Khaled Saifullah, Sushil Ranjan Howlader, Peter Kjær Mackie Jensen

**Affiliations:** 1 Copenhagen Center for Disaster Research, Global Health Section, Department of Public Health, University of Copenhagen, 1014 Copenhagen, Denmark; 2 Institute of Health Economics, University of Dhaka, Dhaka 1000, Bangladesh; 3 icddr,b, Dhaka 1212, Bangladesh; 4 Department of Gastroenterology, Hepatology and Infectious Diseases, University Hospital Düsseldorf, Medical Faculty of Heinrich Heine University Düsseldorf, 40225 Düsseldorf, Germany; 5 Infectious Diseases and Geographic Medicine, Stanford University, Stanford, CA 94305, USA; 6 Bloomberg School of Public Health, Johns Hopkins University, Baltimore, MD 21205, USA

**Keywords:** water, hygiene, Bangladesh, qualitative research, diarrhea

## Abstract

There is a paucity of recent research on direct water quantity measurement for personal and domestic hygiene. We aimed to measure the water quantity used for personal and domestic hygiene and to explore the reasons and determinants for variation of water usage. We conducted this study from September 2014 to June 2016 in a low-income urban community in Dhaka. In 12 households, the team conducted a day-long bimonthly ethnographic observation for one year to measure the volume of water used per activity per person. They conducted 28 in-depth interviews to explore the reasons for changes of water usage. Participants used a median of 75 L (61–100) of water per capita per day (LCPD) and of this 75 LCPD they used a median of 39 LCPD (26–58) for personal hygiene. Women used less water than men. Individual and social norms, beliefs, and weather determinants determined personal hygiene. Water availability determined domestic hygiene (e.g., washing dishes, toilets and bathrooms). This study helps to elucidate a range of determinants of water usage of the participants from the participants’ perspective. The quantity of water used for domestic and personal hygiene and its relationship to fecal-oral transmitted disease can be explored in future research.

## 1. Introduction

The quantity of water used for hygiene plays an important role in diarrheal disease transmission [1,2,3,4]. For example, one study from Nepal showed that people who used inadequate amounts of water per capita per day were at twice the risk of contracting diarrhea than those who used an adequate amount [4]. Diarrheal diseases could spread through use of inadequate water for personal hygiene [5,6,7]. The World Health Organization (WHO) recommends that people with an average use of 20 LCPD water for personal and food hygiene, laundry and bathing are insufficiently equipped to meet basic hygiene needs and are at high risk for health concerns, while 50 LCPD and 100 LCPD of water use are at low risk for health concerns [5,8].

The systematic review of Stelmach and Clasen (2015) examined the relationship between water quantity and health and found that increased water quantity per person was associated with improved health outcomes (i.e., improved trachoma, reduced gastrointestinal infection and diarrheal disease, and improved growth outcomes) [7]. A cross-sectional study conducted among refugee communities in Ghana and Kenya reported that higher amounts of water used for hygiene (per person per day) decreased the risk of diarrhea [9]. An observational study conducted in Malawi found that the incidence of diarrhea in children < 5 years decreased when the amount of water used per person increased [10].

Available studies that measured water quantity were commonly focused on rural and urban [7], semi-urban [11], and refugee communities [6]. The studies reporting water quantity of the residents of a slum area remained limited [12]. WHO recommendations on water quantity and health concerns was first published in 2003 [5], and the second edition was published in 2020 describing similar recommendation using secondary review [8] and to date, methods of measuring water quantity in non-metered water users’ community remains scanty [13]. Climate change is predicted to affect water availability and accessibility which could subsequently exacerbate diarrheal disease. Thus, it is important to generate more evidence on the quantity of water used by urban low-income communities, which forms over half (55%) of the total urban population [14] and who are at high risk of diarrheal diseases and climate vulnerability.

A clear understanding of water use patterns is critical to design efficient and effective water use strategies to combat diarrhea and to develop climate resilient communities. The aim of this study was to measure the water quantity used for personal and domestic hygiene and to explore the reasons behind changes in practices of water usage due to different determinants, such as different months, frequencies, availability, volumes, perceptions and sources.

## 2. Methods

The detail methodology of this study is provided elsewhere, thus we include a brief description here [15,16].

### 2.1. Study Sites

We selected the East Arichpur area of Tongi township in Dhaka, Bangladesh, and collected data from September 2014 to June 2016. East Arichpur is a low-income urban community with an approximate population of 55,504 living within <1 km^2^ [15,16]. Most of the households used on-plot ‘improved’ water sources [15].

### 2.2. Selection of Study Households

This study was part of a larger longitudinal study where 477 households were randomly selected to identify risk factors for diarrhea [15]. To purposively select a subset of households for in-depth exploration, a team of anthropologists roamed within the community for the first two months and conducted several small group discussions and individual informal discussions, to gain understanding of existing water management structure and usage practices among the residents. From this exploration, the team found households with different hours of water access, i.e., for 24 h and <24 h (water available for 6–15 h). Furthermore, the team were able to visit 260 households out of 477 to understand the willingness, availability of the household residents to participate and presence of adult members in the households, who could provide information during data collection. Based on these criteria, the research team selected 24 households to achieve the study objective. Further detailed method of household selection is available elsewhere [15,16]. During this selection process, the team excluded those households who planned to move within the following six months, since it would interrupt one-year data collection procedures. Considering all these characteristics, the team selected 24 households to cover differences in community residents’ perception and practices that can be attributable to differences in water usage in terms of availability and seasonality.

After the selection of study households, the research team spent an average 6 to 8 h per day in the study households for the first two weeks to build a good rapport, gain the trust of the participants and to learn about their daily life and activities. The research team carried a digital recorder and a camera to record events and to make the study participants familiar/habituated with these instruments. They used an ethnographic approach for observation and in-depth interviews to understand the insider/local perspective for water usage of the participants of the study households.

### 2.3. Observation

From the 24 study households, the team selected 12 households based on the availability and willingness of the participants to allow us to perform bi-monthly daylong observation for one year to record water usage for 24 h. The research team conducted 12 to 14 h (from early morning to night) of observation to measure the water quantity used by the household members within 24 h, in each of the 12 households in every other month starting from May 2015 and ending in March 2016. During observation, the team measured the quantity of water used for each type of personal and domestic activities of each member of the households [15] and frequency of water use for personal hygiene. To measure the volume of water, the team used a measured bucket and/or a measured mug before/after the activities. To measure volume for the activities using running water, the team used a stopwatch [15].

### 2.4. In-Depth Interviews

The research team planned to conduct 36 in-depth interviews: 24 with the women of the 24 households and 12 with the men of the 12 observed households. They conducted the in-depth interviews in-between the observations. The team collected information on individual/personal water use practices including frequency and quantity of each activity, the same as recorded in observation. They also explored the reason behind changes in practices due to different months, different frequencies, volumes and sources.

Since data saturation is important to ensure the adequacy of sample size in qualitative research [17], the team continued data collection until reaching data saturation.

### 2.5. Data Analysis

All the in-depth interviews were transcribed verbatim in Bengali. The team expanded all the field notes from observation. The team performed thematic analysis that captured the findings related to research objectives and identified the response patterns within the data set [18]. We (RS, STS and SK) separately reviewed the transcribed data and expanded field notes to identify preliminary codes. After identifying initial codes, we sat together to develop a more comprehensive code list considering the study objectives. The first author reviewed and combined all the coded data in different themes and subthemes to identify similarities and patterns and summarized the findings. Later on, she made comparisons and triangulations among the observational findings, in-depth interviews and field notes of informal conversations, since triangulation is important for the rigor of qualitative research in the coming decades [19].

We calculated the frequency and percentage for categorical data, and median with inter-quartile range (IQR) for the asymmetric distribution of numerical variables. To show the difference of water used between religious performers and non-performers, RS excluded children <5 years old, as they were not expected to perform prayer in Islam.

## 3. Results

The team completed 71 days of observations, 20 in-depth interviews with women, and eight with men, which was less than what was planned in the methods section. This was due to unavailability and unwillingness of participants, and the migration of the households. The team observed a total of 262 days of activities of 59 participants in 15 households (we replaced three households, as they left the study area after two visits). All our study participants were Muslims and more women participated than men (Table 1). Households consisted of a median of 4 members (IQR: 3–5). Over half (52%, 12/23) of those who were involved in income-generating activities, worked in the garment industry or at a factory.

### 3.1. Water Usage for Personal Hygiene

Participants used a median of 39 LCPD of water for personal hygiene (Table 2). Among adults and the age group of 6–17, their water usage varied from drinking, bathing and face washing with soap) (Table 2). Ablution is the ceremonial act of washing parts of the body by Muslims before performing religious prayers [16]. Participants used 72% of the median volume of LCPD for bathing to maintain their personal hygiene. They bathed 91% (238/262) of the person/day during the 262 person/day observation, and 22% (13/59) reported bathing twice a day. It should be noted that the usual practice of taking bath in a low-income community means taking water from a bucket with a mug and pouring the water over one’s head thus drenching the whole body. Participants rinsed their face (using only water) at least once (range: 1–6) in the morning while brushing their teeth and used a median of 2.2 L of water per rinse. Participants provided different rationales for maintaining personal hygiene (Box 1).

Box 1 **Quotations on personal hygiene from the participants of the low-income urban community of Arichpur, Dhaka, from September 2014 to June 2016.**

**Water usage for personal hygiene**

***Feeling of dirt***

*“Since I came from outside (from office, works in a factory), I rinse my hand. I have worked on many desks (of office) and did many tasks, touched many people, so I rinse my hands and face. Sometimes it happened that someone was sweating, and I touched him.”—A male participant*

***Feeling fresh***

*“It feels good and fresh if I use face cream or lotion. In summer, I sweat after sweeping the rooms and don’t feel good. I wash my face to remove the sweat and use some powder. Then I feel better and fresh.”* —*A female participant*

***Maintaining etiquette***

*“I washed my hands and face with soap and water… I am a shopkeeper, I need to look fresh. Every place has some sort of etiquette, hasn’t it?”—A male participant*

***Reasons for not using soap***

*“I did not use soap after defecation. I just used soil to wash it. Why would I lie (to you)? The house I previously rented had soap on the wall of the toilet and had a tube well. After defecation, I used to use soap for handwashing inside the toilet. The environment here (i.e., of this house) is not good. No one keeps soap in the toilet. Here, I follow the practices that everyone does.”*—*A male participant*

*“[Why did you wash hands with soap after cleaning child feces in the morning but did not use soap for handwashing now?] Wasn’t it like loose stool (affirmative)? It was a loose stool, so as soon as I poured water from the pot on it, it washed out. But the morning one (stool) was sticky and smeared in (my) hands too. This one (stool) was almost watery, as soon as I poured water, it washed out (that is why she did not use soap).”*—*A female participant*

***Reasons for not using water after urination***

*“I do not feel good to use water (after urination), so I do not take water. After waking up in the morning, I go to the toilet while rubbing my eyes, I never take water. I never used water, that is why I do not like taking it.”*—*A male participant*

The team observed 41% (24/59) of the participants washing their faces with soap at least once. Although men used more water than women to wash their faces, women (47%, 26/34, 40 times, averaged 3 times per person) washed their faces with soap more frequently than men (32%, 8/25, 21 times, averaged 3 times per person). A higher percentage of young participants washed their faces with soap than adults, as the young participants were more concerned about beautification than adults (Box 1).

Almost all the participants commonly rinsed their hands before and after eating, on average five times per person/day (range: 1–17). Per rinsing episode, they used 0.2 L of water (adults 0.1, children ≤ 5 L). They washed their hands with soap twice (median: 1, range: 1–7) per person/day with an average of one liter of water per episode. Men used more water for handwashing per person/day than women (Table 2), while women (6 times, 20/34) washed hands more frequently than men (4 times, 20/127).

Our team observed that participants washed their hands with soap after 63% (119/190) of the defecation episodes. A total of 56 participants reported defecating during observation. After defecation, 21% (12/56) always washed their hands with soap, 41% (23/56) never used soap for handwashing, and 38% (21/56) occasionally used soap (Box 1).

Participants used more water per episode for cleaning the anal and genital areas after defecation than urination (average 1.8 vs. 0.9 L). Children ≤ 5 years old used 0.2 L per episode after urination. However, three girls aged 6–17 (n = 11) and six children ≤ 5 years old did not use water after urination. Using no water after urination was mainly a practice of men, 50% (8/16) of adult men and 71% (5/7) of men aged from 6–17 usually did not use water, they also provided reasons for not using water after urination (Box 1).

Men used more water than women (median 45 LCPD vs. 38 LCPD) and more men used water outside the home than women (21/24 vs. 19/34). However, adult women used a higher volume of water than adult men (median 52 vs. 48 LCPD) because men commonly avoided using water after urination.

### 3.2. Variation of Water Used for Personal Hygiene Based on Access to Water

**Figure 1:**
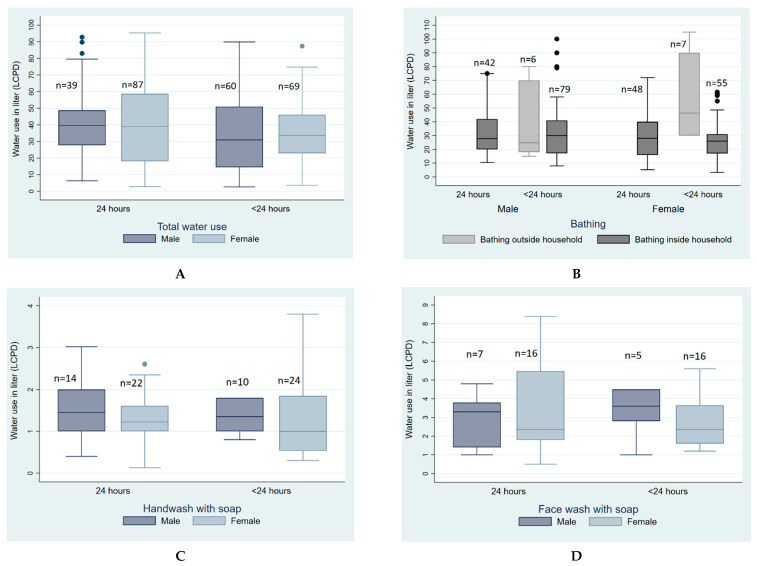

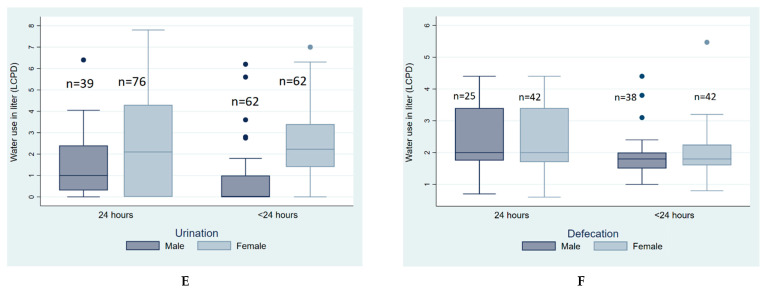
Quantity of water used for personal hygiene LCPD based on water availability among low-income urban residents of Arichpur, Dhaka, from May 2015 to March 2016. (**A**). Total water use, (**B**). Water use for bathing, (**C**). Water use for handwash with soap, (**D**). Water use for face wash with soap, (**E**). Water use for urination, (**F**). Water use for defecation.

Participants with access to water 24 h a day used more water (48 LCPD) than the participants who had <24 h access to water (38 LCPD) in the households (Table S1). Water used for defecation did not change noticeably due to accessibility. Participants with <24 h access tend to perform bathing outside their household as a way of coping with water stress (Figure 1). The participants also reported different coping strategies to deal with water stress (Box 2A).

### 3.3. Variation of Water Usage for Personal Hygiene by Different Months of the Year

**Figure 2:**
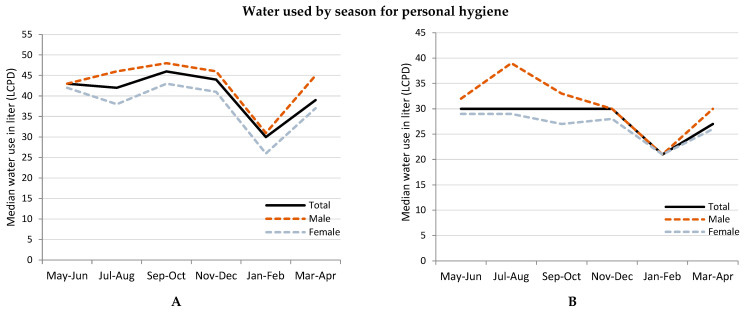

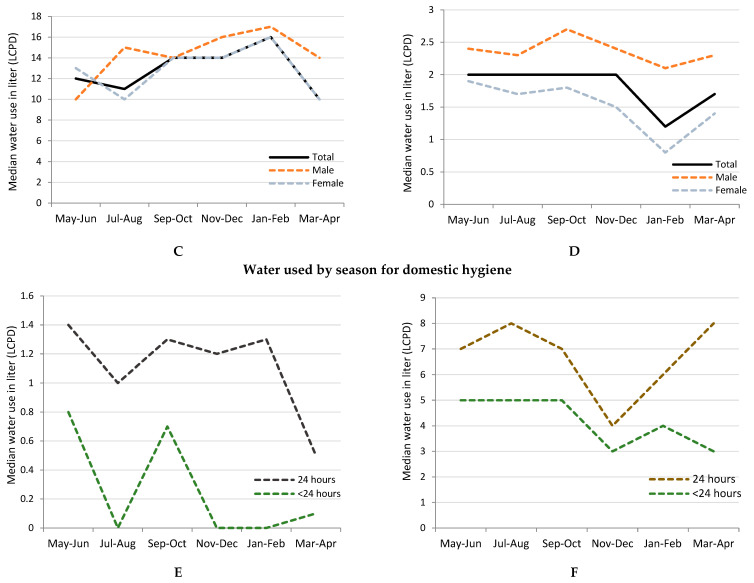
Quantity of water used, LCPD in different months of the year among low-income urban residents of Arichpur, Dhaka, from May 2015 to March 2016. (**A**). Total water used by months, (**B**). Bathing by months, (**C**). Handwashing by months, (**D**). Drinking by months, (**E**). Room(s) cleaning by months, (**F**). Dishwashing by months.

People reduced median water usage for personal hygiene in January and women reduced water usage more than men (Figure 2). The average temperature of January, a winter month with dry weather, was 19 °C and the summer month of May was 30 °C. Participants reported that they did not bathe every day during winter but took baths more than once during summer (Box 2B).

Participants reduced drinking water by almost half in January than in other months (Figure 2). Women drank less volume of water than men (Figure 2). In May, the team observed that participants drank oral rehydration solution (locally known as saline) water, which is usually prescribed for diarrheal patients (Box 2B).

Participants almost used no water for cleaning their sleeping and living rooms between November to April. Their water usage for dishwashing reduced noticeably in November-December among the <24 h water access households (Figure 2).

Box 2 **Quotations from the participants on their personal hygiene based on different determinants in the low-income urban community of Arichpur, Dhaka, from September 2014 to June 2016.**

**A. Water used for personal hygiene based on access**

*“I used to skip bathing sometimes and also did not wash clothes due to water problem… I had to skip bathing sometimes for two days. This happened when there was a water problem. Sometimes, in winter, I had to skip bathing for 8–10 days so that I can use that water for other purposes.”—A female participant*

*“We use this rainwater (for dishwashing and bathing), since we have ‘measured’ (i.e., limited) water and we have to go far away (to collect water) from our room… The rainwater flows down in front of the household door over the corrugated tin roof.”—A female participant*

*“I take a bath in that house (neighboring house). I take my son’s clothes and wash them over there. My granddaughter also goes to that house and wash our clothes…we go to wash clothes in the late afternoon, usually, as no one is there working (in the water point) in the late afternoon.”—A female participant*

**B. Water used for personal hygiene based on different months of the year**

*“I cannot pour more than one bucket of water (because the water gets cold) on my body. Yesterday, I did not even take a bath due to the cold; my youngest daughter also did not take a shower yesterday.”*—*A female participant*

*“I took bath twice for 3–4 days about a week ago because it was very hot. His father (her husband) did the same, though he usually takes showers twice a day during summer.”—A female participant*

*“(My husband) cannot pour more water on his body during winter, as his throat swells. If he catches a cold and coughs, then his throat hurts more.”*—*A female participant*

*“Yes, there is saline prepared in the fridge. When I feel thirsty, I drink the saline from the bottle. I drink it because of the heat/summer. [Do you drink saline in winter?] No. Saline consists of salt concentrations. As people say, drinking salt water can make the body healthy. Hence, I drink saline just because of the heat, since I sweat a lot. If I go to the kitchen or just sit in the warmth, my body sweats a lot. The sweat comes out of the body, right? That is why I drink saline water.”*—*A female participant*

*“Is it possible to drink more than two sips of water during winter? In summer, it is possible to drink more than three sips. I feel like drinking more water since in summer, it feels good. In winter, teeth get sensitive, and stomach churns, so you cannot drink enough water (because water gets cold).”*—*A female participant*

**C. Water used for personal hygiene for religious prayer**

*“I cannot pour more than one bucket of water (because the water gets cold) on my body. Yesterday, I did not even take a bath due to the cold; my youngest daughter also did not take a shower yesterday.”*—*A female participant*

*“People perform ablution to stay holy and pure for regular prayer (namaj). Otherwise, people take bath only once a day, it is not possible to take bath five times in a day. Therefore, ablution is the solution to be holy. Moreover, if a Muslim man keeps himself always in a holy state, it is good for him. No one can say when death will come (the prevailing belief among Muslims is that if a person dies in a holy state, he/she is believed to go to heaven after death). That is why I think it is best to keep oneself in the holy state all the time.”—A male participant*

### 3.4. Variation of Water Usage between Performers and Non-Performers of Regular Prayers

**Figure 3:**
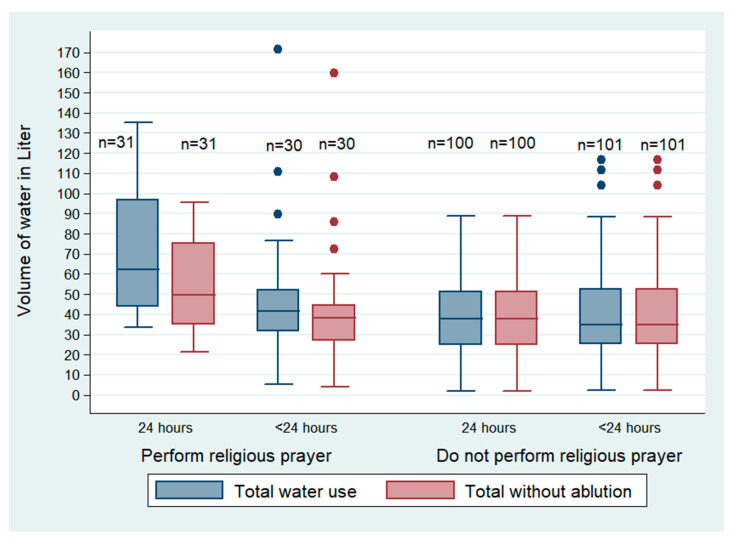
Water used, LCPD) for personal hygiene between religious performers and non-performers by water availability of the low-income urban residents of Arichpur, Dhaka, from May 2015 to March 2016.

Our team observed that the participants who performed regular prayer as a part of Muslim religious practices used more water (both for households with 24 h and <24 h water access) in their daily life, than those who did not perform regular prayers (Figure 3). The adult men and women reported that they tried to keep themselves in a holy state most of the time by performing ablution and washing their feet and hands (Box 2C). Participants who performed regular prayer used 15% (6/39 LCPD) of the total volume of water for ablution (Table 2).

### 3.5. Water Usage for Domestic Hygiene Based on Access to Water

A household used an average of 270 L of water per day. The participants with access to water for 24 h used more water per day for domestic hygiene than those with <24 h access to water (Table 3). They (<24 h access) did not use water to clean rooms and used the least water for cleaning the toilets and bathrooms (Table 3).

“*Sometimes I pour water in the toilet, sometimes I don’t. If it’s dirty, then I pour water, if feces are smeared on the squatting pan.*”

—A male participant

“*Nowadays I need to pour water in the squatting pan. In my previous house the toilet was far away. I used to carry one ‘bodna’ (pot) of water. I never carried more water, as I was lazy. None (of the compound) used to do that. Now that the toilet is close to the room, if I don’t pour enough water, won’t it smell bad (positive sense)?*”

—A female participant

“*Don’t you understand it is somebody else’s house? I pour more water (to make the toilet clean) so that no one can blame me.*”

—A male participant

## 4. Discussion

This study estimated that a person in the study community used an average of 75 LCPD of water, including an average of 39 LCPD of water used for personal hygiene. A person’s age, sex, social norms, beliefs, weather determinants, and water availability determined the quantity of water used for different activities. Earlier studies only attempted either estimating the quantity of water used [11,12,20,21], or its relation to distance, availability, and accessibility [22,23]. In conjunction with the estimation of water quantity, this study depicted insights on participants’ individual reasoning behind the quantity of water used for a particular activity following an ethnographic approach.

We observed that women used somewhat less water than men throughout the year for personal hygiene and used less water than men for washing their hands and face. However, women used more water for specific activities, such as urination, whereas many of the men avoided using water after urination. Water used for specific activities (e.g., face washing) is also associated with shigellosis and trachoma [7]. Thus, both men and women may be at higher risk of contracting different water-related diseases. Furthermore, the findings also suggest that the embedded gender roles and the prevailing norms have important contributions to water usage practices. Using less water not only increases the vulnerability of women to different water borne/related diseases, it also increases the risk of child diarrhea as mothers are responsible for caring for the children and the mother’s hygiene practices are associated with child diarrhea [24,25].

Similarly, religious prayer inspired participants to use extra water to ensure their body was cleaned properly for praying despite limited availability of water. This suggests that religious practices tended to improve and maintain good hygiene as a person devoted to religion is supposed to maintain scrupulous personal hygiene [26]. Therefore, beyond water availability, there are other motivating and social factors influencing the water quantity used for personal and domestic hygiene.

Among these study participants, the water availability changed the volume of water for some specific sanitary and kitchen hygiene activities: toilet and bathroom cleaning, and washing dishes and cooking utensils. The average amount of water used for cleaning toilets was below one LCPD and almost no effort for cleaning of rooms of the house was observed among the households with <24 h water access. In addition, the changes in water usage due to seasonality can contribute to personal hygiene. The quantity of water for washing dishes reduced noticeably in households with <24 h water access compared to households with 24 h water access and in the months of November-December, the cold months of the year. Using a minimal amount of water for sanitation and washing cooking utensils might contribute to spreading fecal-oral transmitted diseases, since the detection of diarrheal pathogen including *V. cholerae* was identified frequently in food plates [27] and drinking glasses and mugs [28,29] in this low-income urban community in Bangladesh. Promotion of handwashing and dishwashing could significantly reduce hand contamination and diarrheal diseases [30].

Water availability had less influence on water usage for face washing. Rather, peer pressure such as social etiquette and appearing ‘fresh’ was an influencing factor and following the same practices as neighbors. Similarly, use of social networks to bathe in neighboring households and use of rainwater were the coping strategies used for bathing among the households who do not have water available for 24 h, as some individual traits such as sweating, touching a sweaty person, taking baths frequently due to sweating and the discomfort felt in hot weather were important drivers for bathing. Curtis et al. (2009) noted, in a review of formative research studies from 11 countries, that social and individual norms, beliefs, habits, and social etiquette affect the handwashing behavior of individuals [31]. Similarly, our study suggests that social and individual factors were important drivers for determining water usage for both personal and domestic hygiene.

The water usage for personal hygiene including frequency and volume of water used for bathing, reduced significantly during the winter month of January compared to the summer month of May among the study participants, which concurs with a study conducted in a low-income community in Mumbai, India, with relatively similar weather patterns to Dhaka [12]. The reduced use of water in the winter months coincides with the peak incidence of rotavirus diarrhea in Bangladesh [32]. The relationship between water borne/related diseases and the season is well noted in the research, but the relationship between seasonal water usage for personal and domestic hygiene and its relationship to diseases was little explored. Hence, future studies on diarrhea and seasonality, particularly for rotavirus, may consider an investigation of water quantity used for children and the incidence of disease/infection.

The average water used for this study’s participants was 75 LCPD, including water for personal and domestic hygiene, which was in between the basic water requirement of 50 LCPD [5,33] and the optimal access of 100 LCPD suggested in the WHO guideline [5]. The use of 28 LCPD water for bathing was similar to the findings of 27 LCPD of the study conducted in rural villages of Bangladesh [34] and in urban settings of Uganda, located in the tropical climatic zone [20]. The average water used for the toilet and bathroom was one LCPD, and the average water used for dishwashing was five LCPD, both of which are below the basic required service level suggested by Gleick (1996) and may, therefore, pose health concerns [33].

Most policy guidance reports and intervention strategies [5,35,36,37] in the last three decades emphasized water access and sanitation facilities within household premises, as this was presumed to increase water usage for personal and domestic hygiene. The WHO guideline on ‘domestic water quantity, service level and health’ published in 2003 described the availability and accessibility issues, and critically discussed the necessity of ensuring water source at dwellings and within the household to enhance personal and domestic hygiene and thereby reduce hygiene-related health risks. The target and indicators of Sustainable Development Goal (SDG) 6 to ‘ensure availability and sustainable management of water and sanitation for all’ [37] and goal 11 to ‘make cities and human settlements inclusive, safe, resilient and sustainable’ emphasized ensuring equitable access for drinking water, handwashing facilities, and sanitation facilities at home, and upgradation of slums by ensuring access for all to adequate, safe and affordable housing and basic services [38]. Ensuring pipe-to-plot water sources was also emphasized as part of the targets of the SDGs. However, considering these study findings, we would argue that ensuring in-plot water availability is an important driver for improved hygiene, but availability only cannot ensure improved hygiene practices unless social norms, individual traits and motivating factors are considered. For example, individual physical comfort (e.g., feeling and looking fresh) and social norms that support religious rituals/rules could be used as motivating factors to improve hygiene practices through public health interventions. Incorporating such non-infrastructural determinants can be beneficial to understand the diarrheal disease risk and to determine where to focus efforts to combat water-related diseases in future climate change and water stress situations.

One limitation of this study was the risk of observation bias. Participants may have exaggerated their water use practices for different activities in the presence of the observer. However, to minimize the observation bias, the research team spent more time with the participants of the study household before the actual observation started. Moreover, the observation method was more reliable than the interview, since it did not influence the participants to recall their water use [39]. There was no known comprehensive method to measure water use for non-metered water use areas [7], which is prominent in low-income countries. It is possible that practices could be different in other households within the same communities or in other communities. However, the findings of this study were consistent with the findings of other studies [12,20,34]. This study was conducted using a small sample size following qualitative research design, and thus, the findings of this study may not be generalizable particularly to other locations, e.g., emergency settings including disaster relief settings and arid locations with extreme water shortage. Nevertheless, the findings will be useful for the similar low-income urban settings, which is growing faster as part of urbanization.

## 5. Conclusions

This study helps to elucidate a range of determinants of water used for personal and domestic hygiene from the perspective of the participants and argues that behavioral and social determinants should be incorporated in water and hygiene related targets and indicators. Future studies can examine if religious motivation of cleaning reduces diarrheal diseases. The minimum water quantity usage for sanitary (use of water for toilet and bathroom cleaning) and kitchen hygiene (water used for dishes) and its relation to fecal-oral transmitted disease should be explored in future research. Since there is a paucity of epidemiological research on direct water quantity measurement and no standard or validated approach exists for measuring water quantity in the non-metered water user community, incorporating this observation method might be useful to develop and validate water measurement methodology for a large sample in the future.

## Figures and Tables

**Table 1:** Profile of the study household residents of the low-income urban community of Arichpur, Dhaka, from May 2015 to March 2016.

Characteristics of Study Households	Number of Households n (%)
Total households	25
Households selected for observation	15 (60)
Households selected for in-depth interviews	24 (96)
Availability of water	
24 h availability *	13 (52)
<24 h availability	12 (48)
Average household members	4
**Characteristics of Observed Household Members**	**Number of Persons**
Total participants	59
***Gender***	**(n = 59)**
Male	25 (42)
Female	34 (58)
***Age***	**(n = 59)**
Adults (18+)	34 (58)
Aged 6–17	18 (31)
Children ≤ 5	7 (12)
***Religion***	**(n = 59)**
Muslim	59
***Not involved in earning***	**(n = 36)**
Student	16 (46)
Housewife	9 (25)
Children	8 (22)
Unemployed	3 (8)
***Involved in earning***	**(n = 23)**
Garment/factory worker	12 (52)
Small business	4 (17)
Service holder	3 (13)
Day labors	3 (13)
Beggar	1 (4)
***Education***	**(n = 59)**
Children not eligible for education	6 (10)
No education	13 (22)
Primary education	17 (29)
Secondary education	19 (32)
Higher secondary education	1 (2)
Graduate	3 (5)

* One household did not participate in the in-depth interview.

**Table 2:** Volume of water used for personal hygiene among the low-income urban residents of Arichpur, Dhaka, from May 2015 to March 2016.

Water Used for Personal Hygiene-Liter per Capita per Day (LCPD)								
	Adult		Age 6–17 Years		Age ≤ 5 Years		All Respondents	
Activities	Median	(IQR)	Median	(IQR)	Median	(IQR)	Median	(IQR)
Total for personal hygiene	50	(36–68)	36	(21–46)	15	(9–19)	39	(26–58)
Drinking	2.4	(1.9–3.3)	1.3	(0.9–1.7)	0.5	(0.4–0.9)	1.9	(1.1–2.7)
Bathing	35	(24–48)	26	(17–31)	9	(8–15)	28	(18–40)
All activities excluding drink and bath	13	(8–21)	9	(6–12)	4	(2.6–6)	10	(6–16)
Face rinse	2.6	(1.8–4.5)	2.1	(1.4–4.1)	1.3	(0.5–1.9)	2.2	(1.6–4.2)
Face wash with soap	3.3	(2.2–7)	2.5	(1.7–4.2)	1.6	(1.4–1.9)	2.5	(1.8–4.5)
Hand rinse	1	(0.6–1.9)	0.7	(0.4–1.2)	0.3	(0.2–0.5)	0.9	(0.5–1.6)
Hand wash with soap	1.5	(1–2)	1.3	(0.6–1.6)	0.4	(0.1–0.7)	1.3	(0.9–2)
Feet rinse	1.3	(0.9–2.4)	1.2	(0.8–1.8)	1.2		1.2	
Face, hand and feet rinse/wiping body	3.2	(2.6–6)	3.4	(1.8–4.2)	1.8	(0.6–3)	3.3	(2.6)
After defecation	2	(1.7–2.8)	1.9	(1.7–2.2)	2.2	(1.5–3.7)	2	(1.7–2.5)
After urination	2.8	(1.5–5.1)	2.5	(1.3–3.9)	0.4	(0.2–0.6)	2.6	(1.5–4.6)
Ablution*	7.8	(3.6–19)	2.5	(1.4–6.7)			6.1	(2.9–14.9)
**Water Used by Gender**								
	Adult Man		Adult Woman		Man, Age 6–17		Woman, Age 6–17	
Total for personal hygiene, all ages	45	(27–58)	38	(25–56)				
Total for personal hygiene	48	(32–68)	52	(38–67)	39	(22–47)	35	(21–44)
Drinking	2.7	(2.1–3.5)	2.1	(1.6–3)	1.6	(1–2.2)	1.2	(0.9–1.6)
Bathing	34	(21–48)	35	(25–45)	27	(15–34)	26	(18–30)
All activities excluding drink and bath	11	(8–20)	15	(11–22)	10	(6–14)	8	(6–12)
Face rinse	2.9	(2–5.3)	2.2	(1.6–4.4)	2.2	(1.4–4.3)	2.1	(1.5–4)
Face wash with soap	3.5	(1.8–16)	2.7	(2.2–5)	3.8	(2.8–4.5)	2.3	(1.4–3.6)
Hand rinse	0.9	(0.6–1.5)	1.3	(0.8–2.3)	0.8	(0.4–1.5)	0.7	(0.4–1)
Hand wash with soap	1.7	(0.9–2)	1.4	(1–2.2)	1.4	(1–2.1)	1.1	(0.5–1.6)
Feet rinse	1.2		1.5		1.4		0.9	
Face, hand and feet rinse/wiping body	4		3		3.6		2.2	
After defecation	2	(1.7–2.8)	2	(1.7–2.8)	1.8	(1.5–2.4)	1.9	(1.7–2.1)
After urination	1.6	(1–2.9)	3.8	(2–6.3)	1.7	(1–2.8)	2.6	(1.6–4)
Ablution *	10	(4–27)	8	(3.6–12)	10	(3–13)	1.4	(1.3–2.8)

* The ceremonial act of washing parts of the body by Muslims before performing religious prayers.

**Table 3:** Volume of water used based on water availability for domestic hygiene among the low-income urban community residents of Arichpur, Dhaka, from May 2015 to March 2016.

Activities	Water Used per Household (LHPD *)		Water Used per Person (LCPD ^†^)	
	Median	(IQR)	Median	(IQR)
**Total personal and domestic water used**	270	(227–360)	75	(61–100)
24 h	318	(240–374)	79	(71–118)
<24 h	253	(198–310)	65	(57–89)
**Cleaning dishes**	18	(12–26)	5	(3–8)
24 h	23	(14–31)	7	(4–9)
<24 h	15	(9–20)	4	(3–6)
**Cleaning clothes**	62	(37–90)	15	(9–28)
24 h	68	(44–91)	18	(12–28)
<24 h	51	(18–80)	10	(5–31)
**Cleaning sleeping and living room**	1.5	(0–7)	0.4	(0–2)
24 h	4	(0–8)	1	(0–2)
<24 h	0	(0–4)	0	(0–1)
**Cleaning toilet and bathroom**	4	(1–11)	1	(0.4–3)
24 h	11	(4–18)	3	(1–5)
<24 h	2	(0–6)	1	(0–2)
**Cooking and food preparation**	19	(13–26)	6	(4–8)
24 h	19	(15–26)	5	(4–8)
<24 h	18	(13–27)	6	(4–8)

* Liter per household per day, which was calculated by dividing the total volume of water with the number of household members in the household. ^†^ Liter per capita per day.

## Data Availability

The data presented in this study are available on request from the corresponding author. The data are not publicly available due to privacy issue. The information shared by the participants includes personal and private life activities/issues, and thus, sharing the data publicly is not possible.

## Supplementary Materials

The following supporting information can be downloaded at: https://www.mdpi.com/article/10.3390/ijerph192315656/s1, Table S1: Comparative analysis on water used for personal hygiene considering availability of water among low-income urban residents of Arichpur, Dhaka from May 2015 to March 2016; Table S2: Quantity of water used liter per capita per day (LCPD) in different months of the year among low-income urban residents of Arichpur, Dhaka from May 2015 to March 2016.
