# Supplementary material for: Measuring Water Quantity Used for Personal and Domestic Hygiene and Determinants of Water Use in a Low-Income Urban Community
Source: Int J Environ Res Public Health. 2022 Nov 25;19(23):15656. doi: 10.3390/ijerph192315656 (PMC9737866; doi:10.3390/ijerph192315656)
Supplement: Supplementary file 1 [file ijerph-19-15656-s001.zip › ijerph-1908721-supplementary.pdf]

Table S1. Comparative analysis on water used for personal hygiene considering availability of water among low-income urban residents of Arichpur, Dhaka from May 2015 to March 2016

	Water available 24 hours			Water available <24 hours		
**Activities**	P/d*	Frq¶	**Median [IQR]**	P/d*	Frq¶	**Median [IQR]**
Total personal hygiene	131	N/A‡	**48 [25-60]**	131	N/A‡	**38 [27-56]**
Drinking	131	758	**1.6 [0.9-2.4]**	131	875	**2.1 [1.4-2.9]**
Bathing	121	125	**28 [18-40]**	117	126	**29 [18-40]**
All activities excluding drink & bath	131	N/A‡	**11 [6-20]**	131	N/A‡	**10 [7-15]**
Ablution	31	116	**15 [8-23]**	30	50	**3 [2-5]**
Face rinse	115	195	**2 [1.5-4.0]**	113	219	**2.5 [1.8-4.5]**
Face wash with soap	26	36	**3 [1.9-6]**	21	25	**2.5 [1.8-3.7]**
Hand rinse	123	581	**0.8 [0.4-1.4]**	125	624	**1 [0.6-1.6]**
Hand wash with soap	40	76	**1.3 [1-2]**	34	53	**1.3 [0.8-1.8]**
Feet rinse	67	127	**1.1 [0.6-2]**	77	149	**1.4 [0.9-2.2]**
Face hand & feet-rinse/wipe body	27	40	**3.8 [2.6-9]**	32	47	**2.9 [1.8-4.1]**
After defecation	71	93	**2 [1.7-3.4]**	83	97	**1.5 [1.6-2.2]**
After urination	93	342	**3.1 [1.5-5.8]**	93	275	**2.1 [1.4-3.6]**

* P/d= Person/day of water use activities. ¶ Frq= Frequency of water use activities. N/A‡= Frequency is not applicable since it is a cumulative total of all activities

Table S2. Quantity of water used liter per capita per day (LCPD) in different months of the year among low-income urban residents of Arichpur, Dhaka from May 2015 to March 2016

	**Water used for personal hygiene -liter per capita per day (LCPD)**																	
	**Visit 1 (May 2 to Jun 10, 15) Average temp: 30° C**			**Visit 2 (Jul 5 to Aug 1, 15) Average temp: 28° C**			**Visit 3 (Sep 6 to 15, 15) Average temp: 30° C**			**Visit 4 (Nov 1 to 27, 15) Average temp: 35° C**			**Visit 5 (Jan 3 to 28, 16) Average temp: 19° C**			**Visit 6 (Mar 2 to 21, 16) Average temp: 28° C**		
**Activities**	P/d*	Frq¶	**Median [IQR]**	P/d*	Frq¶	**Median [IQR]**	P/d*	Frq¶	**Median [IQR]**	P/d*	Frq¶	**Median [IQR]**	P/d*	Frq¶	**Median [IQR]**	P/d*	Frq¶	**Median [IQR]**
Total personal hygiene	46	N/A‡	**43 [32-74]**	48	N/A‡	**42 [19-60]**	41	N/A‡	**46 [31-52]**	43	N/A‡	**43 [30-56]**	42	N/A‡	**30 [14-43]**	42	N/A‡	**39 [24-59]**
Drinking	46	301	**2 [1.3-2.8]**	48	288	**2 [1.4-2.9]**	41	270	**2 [1.3-3]**	43	277	**2 [1.3-3.1]**	42	223	**1.1 [0.7-2.3]**	42	274	**1.7 [1.2-2.4]**
Bathing	45	50	**30 [20-45]**	42	47	**30 [18-48]**	39	41	**30 [20-40]**	40	40	**30 [17-36]**	33	33	**21 [16-32]**	39	40	**27 [19-42]**
Excluding drink & bath	46	N/A‡	**11 [7-20]**	48	N/A‡	**8 [5-15]**	41	N/A‡	**11 [8-15]**	43	N/A‡	**13 [8-18]**	42	N/A‡	**9 [6-13]**	42	N/A‡	**11 [6-16]**
Ablution	11	25	**4 [2.2-13]**	12	39	**11 [3.4-20]**	9	24	**6.7 [3-10]**	12	29	**3.3 [2-18]**	7	24	**9.6 [4.5-20]**	10	25	**6 [3-16]**
Face rinse	40	83	**2.8 [1.7-5.1]**	35	57	**2.8 [1.6-4.5]**	39	70	**2 [1.5-4.1]**	39	80	**2.9 [1.8-5.7]**	36	55	**1.9 [1.3-2.3]**	37	69	**2.2 [1.6-3.5]**
Face wash with soap	7	7	**1.4 [1.3-2.5]**	7	8	**2.3 [1.4-2.8]**	6	9	**3.3 [1-8]**	12	15	**3.6 [2.3-4.7]**	10	15	**3.8 [2.3-4.5]**	5	7	**2.5 [2.4-8.4]**
Face hand & leg- rinse/ wipe body	14	26	**4 [2.2-11]**	9	11	**2.8 [2.5-4]**	14	23	**3.7 [3-6]**	13	17	**3.5 [1.8-5.7]**	1	1	**3 [0]**	8	9	**2.6 [1.9-2.9]**
Hand rinse	42	205	**1 [0.6-2]**	43	194	**7.5 [0.5-1.3]**	41	174	**1 [0.6-1.5]**	41	210	**1 [0.6-1.7]**	40	202	**0.7 [0.4-1.7]**	41	220	**1 [0.6-1.5]**
Hand wash with soap	14	29	**1.2 [0.8-2.8]**	9	9	**1.1 [1-1.5]**	9	16	**1.4 [1-1.8]**	15	25	**1.4 [1-2.1]**	11	21	**1.6 [0.7-20]**	16	29	**1 [0.6-1.9]**
Leg rinse	21	38	**1.3 [0.9-1.8]**	23	33	**1.3 [0.5-1.5]**	22	42	**1 [0.5-2]**	29	51	**1.1 [0.9-2.2]**	22	52	**1.8 [1.2-2.3]**	27	60	**1.1 [0.6-2.2]**

After defecation	30	40	**2 [1.6-2.5]**	20	23	**2 [1.4-2.2]**	22	27	**2.1 [1.8-3.2]**	27	35	**2 [1.7-3]**	28	32	**1.9 [1.7-2]**	27	33	**2 [1.6-3]**
After urination	33	92	**2.6 [1.4-5.4]**	32	99	**2.9 [1.4-5]**	30	102	**3.7 [1.9-6.2]**	32	119	**2.5 [1.6-5.5]**	30	96	**2 [1.5-2.6]**	29	109	**2.5 [1.8-3.6]**

[*] P/d= Person/day of water use activities. [¶] Frq= Frequency of water use activities. N/A[‡]= Frequency is not applicable since it is a cumulative total of all activities
